# Identification of 5 Gene Signatures in Survival Prediction for Patients with Lung Squamous Cell Carcinoma Based on Integrated Multiomics Data Analysis

**DOI:** 10.1155/2020/6427483

**Published:** 2020-06-08

**Authors:** Hongxia Ma, Lihong Tong, Qian Zhang, Wenjun Chang, Fengsen Li

**Affiliations:** Pneumology Department, The Traditional Chinese Medicine Hospital Affiliated to Xinjiang Medical University, Urumqi City, China

## Abstract

**Background:**

Lung squamous cell carcinoma (LSCC) is a frequently diagnosed cancer worldwide, and it has a poor prognosis. The current study is aimed at developing the prediction of LSCC prognosis by integrating multiomics data including transcriptome, copy number variation data, and mutation data analysis, so as to predict patients' survival and discover new therapeutic targets.

**Methods:**

RNASeq, SNP, CNV data, and LSCC patients' clinical follow-up information were downloaded from The Cancer Genome Atlas (TCGA), and the samples were randomly divided into two groups, namely, the training set and the validation set. In the training set, the genes related to prognosis and those with different copy numbers or with different SNPs were integrated to extract features using random forests, and finally, robust biomarkers were screened. In addition, a gene-related prognostic model was established and further verified in the test set and GEO validation set.

**Results:**

We obtained a total of 804 prognostic-related genes and 535 copy amplification genes, 621 copy deletions genes, and 388 significantly mutated genes in genomic variants; noticeably, these genomic variant genes were found closely related to tumor development. A total of 51 candidate genes were obtained by integrating genomic variants and prognostic genes, and 5 characteristic genes (HIST1H2BH, SERPIND1, COL22A1, LCE3C, and ADAMTS17) were screened through random forest feature selection; we found that many of those genes had been reported to be related to LSCC progression. Cox regression analysis was performed to establish 5-gene signature that could serve as an independent prognostic factor for LSCC patients and can stratify risk samples in training set, test set, and external validation set (*p* < 0.01), and the 5-year survival areas under the curve (AUC) of both training set and validation set were > 0.67.

**Conclusion:**

In the current study, 5 gene signatures were constructed as novel prognostic markers to predict the survival of LSCC patients. The present findings provide new diagnostic and prognostic biomarkers and therapeutic targets for LSCC treatment.

## 1. Introduction

The incidence and mortality of lung cancer have been increasing annually all over the world in the past few decades [1], allowing lung cancer to become a leading cause of male cancer death and the second most frequent cause of female cancer death right behind breast cancer [2]. Lung squamous cell carcinoma (LSCC) is the second most common pathological type of lung cancer, second to lung adenocarcinoma. LSCC accounts for 40%-50% of all lung cancer cases, and its early symptoms are not obvious and atypical; thus, most patients are already at a middle or late stage by the time of diagnosis [3]. In recent years, advances in scientific research and clinical practice and achievements have been made in understanding the mechanism of the occurrence and development of lung cancer; moreover, predictive screening indicators and targeted drug therapy have also been improved. However, scientific research and achievements concerning lung adenocarcinoma and LSCC are limited; therefore, regarding such a research gap, the study of LSCC is highly urgent and necessary.

Multiomics data, such as cancer genome mapping (TCGA) and therapies applied to research (TARGET) projects, have the potential to generate effective treatments, as they can be used effectively to predict disease progression [4]. Liu et al. predicted the prognosis of high-risk diseases by integrating the data of copy number variations (CNVs) and single-nucleotide polymorphisms (SNPs) [5]. SNPs account for 1% of the human genomes and exist in coding or noncoding regions that affect exon splicing or transcription [6]. SNPs have been considered predictive markers of complex diseases [7] and have been found to be associated with many common diseases, including type II diabetes [8], Crohn's disease [9], schizophrenia [10], and breast cancer [11]. However, the functional significance and gene/variant alleles of novel disease-related SNPs studied by genome-wide association studies (GWAS) or next-generation sequencing (NGS) data remained a challenge to be investigated. CNVs are defined as sequence variants, which ranged from 50 bps to a few megabits (Mb) in size, and have deletions, duplicates, triplets, insertions, complex genome rearrangements (CGR), and other CNVs [12]. CNVs have more than 10 times differences in heritable sequences compared with single nucleotide variants (SNVs) in the general population [13], and its genome-wide map has been comprehensively studied [14].

Recently, many genetic biomarkers for LSCC patients have been studied, but most of these studies have focused on a single gene in LSCC's prognosis, recurrence, or diagnosis, for example, PD-L1 [15], family with sequence similarity 83 member B (FAM83B), hyaluronidase 3 (HYAL3), and minichromosome maintenance protein 2 (MCM2) [16]. Moreover, in some other studies, more than one gene is identified; for example, a prognostic risk model constructed by 4 abnormally methylated genes (VAX1, CH25H, AdCyAP1, and Irx1) has been found to be able to predict the survival rate of LSCC patients [17]. A previous study established 4-gene expression signature clustering models with 14 genes collected from cluster patient samples and indicated that the signature could effectively help predict the prognosis of LSCC patients and improve treatment strategies [18]; however, this also poses difficulties and challenges in clinical applications, as selecting the suitable signature is not an easy work. Therefore, it is essential to define and validate an effective genetic signature model for predicting LSCC prognosis.

Gene expression profile, single-nucleotide mutation, and CNVs of patients with LSCC were obtained from large datasets in TCGA and GEO databases. Prognostic markers were screened by integrating genomics and transcriptome data to establish a 5-gene signature, and its ability to predict survival was further verified by an internal test set and an external validation set. We found that the 5-gene signature is involved in important biological processes and pathways of LSCC, and similar results were also shown by GSEA analysis, suggesting that the 5-gene signature could effectively predict the prognostic risk of patients with LSCC. Thus, the signature established in the current study could provide a basis for better understanding of the molecular mechanism of LSCC prognosis.

## 2. Materials and Methods

### 2.1. Data Acquisition and Processing

TCGA RNA-Seq FPKM data contained a total of 553 samples, and clinical follow-up information contains 758 samples with SNP chips 6.0; copy number variation data contained 501 samples downloaded from UCSC; mutation annotation information (MAF) contains 178 samples downloaded using GDC client, downloaded from the GEO standardized expression profile; and clinical information contains 176 samples of GSE42127 [19] data; among them, a total of 43 had clinical follow-up information downloaded from GEO, and download date was on June 5, 2019. A total of 741 LSCC cases with follow-up information were collected from TCGA RNASeq data and further randomly divided into a training set (*N* = 247) and a test set (*N* = 494). GSE42127 data with clinical follow-up information served as the external validation set. Sample information of each group is shown in Table 1.

### 2.2. Univariate Cox Proportional Hazard Regression Analysis

Guo et al. [20] previously performed univariate Cox proportional risk regression analysis with the training dataset for each gene to screen genes significantly related to overall survival (OS) of patients; *p* < 0.05 was also defined as the threshold in the present study.

### 2.3. Analysis of CNV Data

GISTIC was widely used to detect both broad and focal (potentially overlapping) recurring events; GISTIC 2.0 [21] software was used to identify genes with significant amplification or deletion according to the parameter thresholds of amplified or absent fragments > 0.1 and *p* < 0.05.

### 2.4. Genetic Mutation Analysis

In order to identify the genes with significant mutations, Mutsig 2.0 software was used to identify the genes with significant mutations in the maf file of TCGA mutation data, with a threshold value of *p* < 0.05.

### 2.5. Construction of Prognostic Gene Signature

Genes significantly associated with patient OS and those with amplification, deletion, and mutation were selected and subjected to random survival forest algorithm to rank genes that showed prognostic values [22]. As previously described by Meng et al. [23], R package random survival forest was used for screening, with the Monte Carlo iteration number set as 100 and the previous progress number set as 5; moreover, a gene with relative importance greater than 0.27 was identified as a characteristic gene. Additionally, multivariate Cox regression analysis was carried out, and the following risk scoring model was constructed:

(1) $$\begin{array}{c} \text{Risk score}=\overset{n}{\underset{k=1}{\sum }}{\text{Exp}}_{k}*{e^{\text{HR}}}_{k}, \end{array}$$

where *n* is the number of prognostic genes, Exp_*k*_ is the expression value of the prognostic genes, and *e*^HR^_*k*_ is the estimated regression coefficiency of genes in the multivariate Cox regression analysis.

### 2.6. Functional Enrichment Analyses

Gene Ontology (GO) and Kyoto Encyclopedia of Genes and Genomes (KEGG) pathway enrichment analysis was performed using the R package clusterprofiler [24] to identify overrepresented GO terms in three categories (biological processes, molecular function, and cellular component) and KEGG pathway. For the analysis, a FDR < 0 .05 was considered statistically significant.

GSEA [25] was performed by JAVA program (http://software.broadinstitute.org/gsea/downloads.jsp) using MSigDB [26] C2 Canonical pathway gene set collection, which contains 1320 gene sets. After performing 1000 permutations, gene sets with a *p* value lower than 0.05 were considered to be significantly enriched.

### 2.7. Statistical Analysis

The Kaplan-Meier (KM) curve was plotted by using the median risk score in each dataset as a cutoff to compare the risk of survival between the high-risk group and the low-risk group. Multivariate Cox regression analysis was conducted to examine whether gene markers were independent prognostic factors. Statistical significance was defined as *p* < 0.05. The AUC analysis was performed using the R package pROC, and the heat map was drawn using the R package pheatmap. All analyses applied default parameters except for special instructions, which are performed in R software version 3.4.3.

## 3. Results

### 3.1. Analysis of Multiomics Data to Identify Genes Associated with Overall Survival of Patients with LSCC

For the samples of the TCGA training set, univariate Cox regression analysis was performed to establish a relationship between OS of patients and gene expression. 804 prognostically significant genes were identified, and information of the 20 genes with the highest significance is shown in Table 2.

### 3.2. Gene Set for the Identification of Genomic Variation

**Figure 1:**
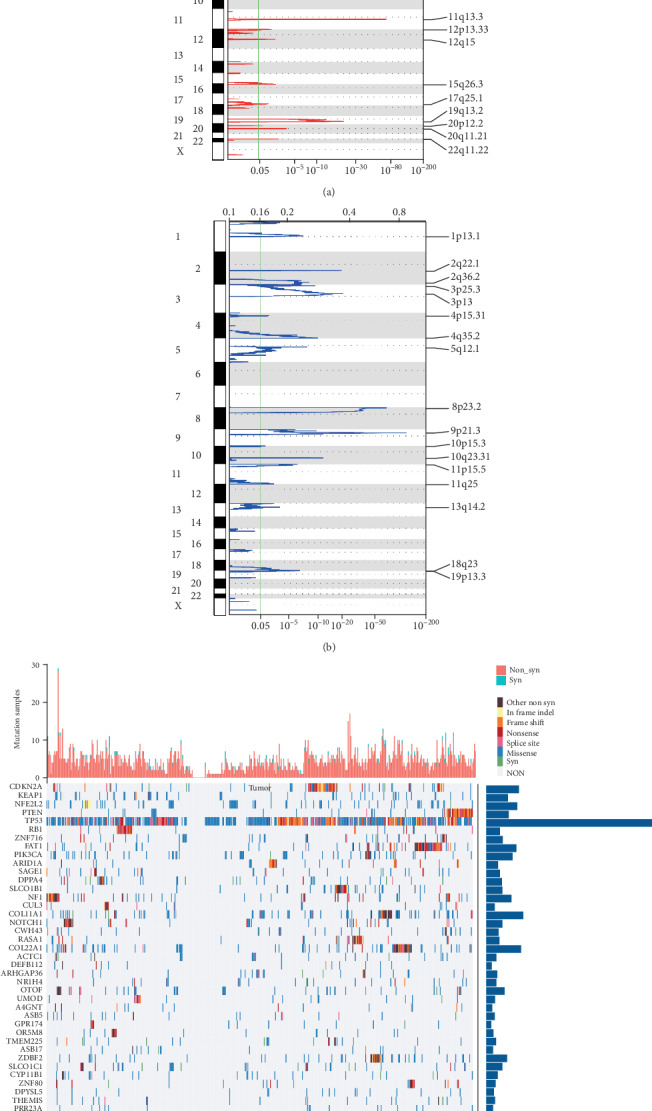
(a) A significantly amplified fragment of lung squamous cell carcinoma genome (LSCC). (b) A significant deletion fragment of the LSCC genome. (c) The distribution of the 50 genes with most significant *p*-valued genes in patients with LSCC; the bar chart at the top shows the total number of synonymous and nonsynonymous mutations in 50 genes in each patient, while the bar chart at the right shows the number of mutations in 50 genes in all samples.

For CNV data in TCGA, GISTIC 2.0 was used to identify genes with significant amplification or deletion, with parameter thresholds of amplification or deletion > 0.1 and *p* < 0.05.  Figure 1(a) shows significantly amplified fragments of the LSCC, and a total of 535 genes were amplified. Among them, EGFR was significantly amplified at the 7p11.2 segment (*q* = 1.33*E* − 16); CD72 was significantly amplified on the 9p13.3 segment (*q* value = 1.38*E*-07); CDK3 was significantly amplified on the 17q25.1 segment (*q* value = 0.0092281).  Figure 1(b) shows the segments of the LSCC genome with significant deletion, and a total of 621 genes were deleted. A significant loss of CDKN2A on the 9p21.3 segment (*q* value = 7.83*E*-116) and a significant loss of FOXP1 on the 3p13 segment (*q* value = 6.47*E*-21) were observed; moreover, RB1 was absent in the 13q14.2 segment (*q* value = 0.0012441). For the TCGA mutation annotation data, Mutsig2 used to identify genes with significant mutations screened a total of 388 genes with significant mutation frequencies. The distribution of synonymous mutations, missense mutations, frame-insertion or deletion, frame-shifting, nonsense mutations, shear sites, and other nonsynonymous mutations in TCGA patients showed the most significant *p* values (Figure 1(c)), and CDKN2A, PTEN, TP53, RB1, PIK3CA, and some other genes were found closely related to the occurrence and development of LSCC.

### 3.3. Functional Analysis of CNV Genes and Mutated Genes

**Figure 2:**
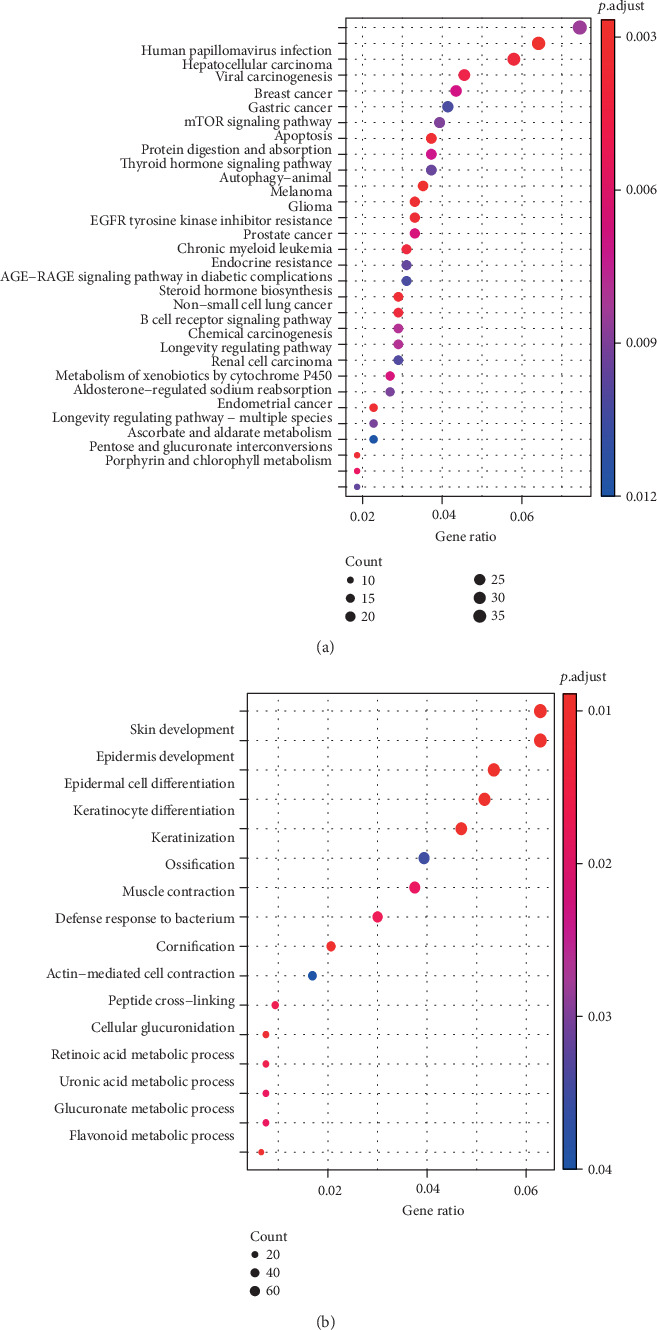
(a) 1385 genes with copy number variation and mutation are involved in the KEGG pathway. (b) Biological processes involve 1385 genes with copy number variation and mutation (GO bp).

In order to analyze the function of genomic mutant genes, a total of 1385 amplified and deleted genes and significantly mutated genes identified were integrated. GO biological process and KEGG functional enrichment analysis were performed on the 2261 genes. The results of KEGG enrichment analysis revealed that the 1385 genes were significantly enriched in the mTOR signaling pathway, cell apoptosis, autophagy, EGFR tyrosine kinase inhibitor resistance, non-small cell lung cancer, B cell signaling pathway, and other KEGG biological pathways related to the development of cancer (Figure 2(a)). In the category of the biological process, the 1385 genes were mainly enriched in epidermal development, epidermal cell differentiation, keratinocyte differentiation, and other GO terms (Figure 2(b)). Noticeably, these terms are also closely related to the occurrence and development of cancer; in other words, these genomic mutations are closely related to tumors.

### 3.4. Identification of a 5-Gene Signature for LSCC Survival

**Figure 3:**
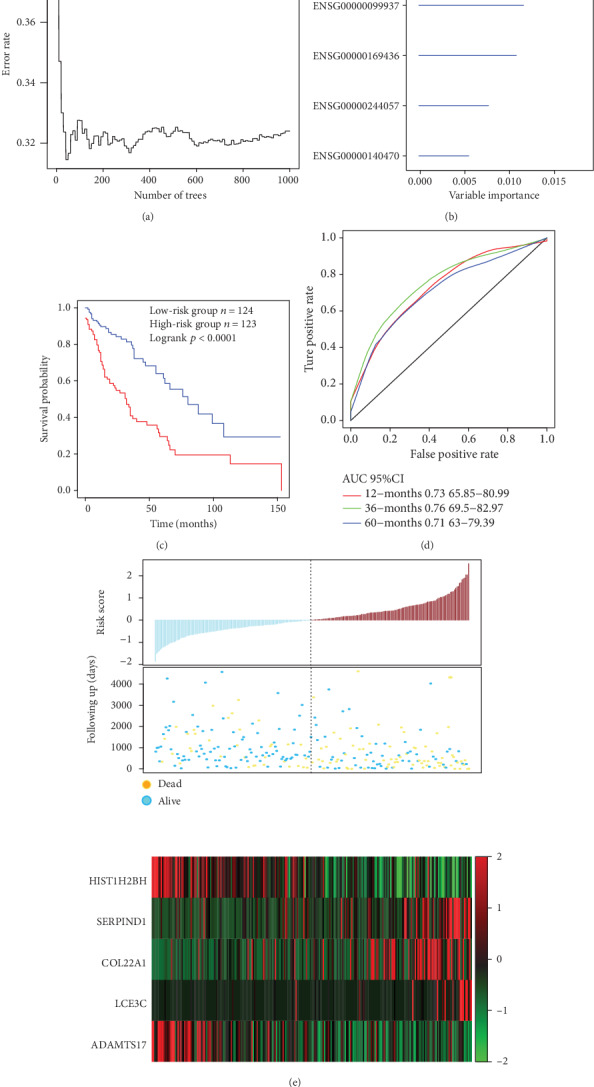
(a) The relationship between the error rate and the number of classification trees. (b) Importance order of 5 genes out-of-bag. (c) Distribution of KM survival curves of the 5-gene signature in the TCGA training set. (d) The ROC curve and AUC of the 5-gene signature classification. (e) Risk score, survival time, survival status, and expression of the 5 genes in TCGA training.

First, a total of 804 candidate prognostic genes of gene variants and prognostic genes were integrated, and we finally identify 51 genes with amplification, deletion, and mutation as candidate genes. Furthermore, a random survival forest algorithm is used for feature selection, and the relationship between the error rate and the number of classification trees is shown in Figure 3(a). Genes with relative importance of > 0.27 served as the final signature, and finally, 5 genes were obtained (Table 3). These genes play important roles in the regulation of tumor-related pathways and biological processes; however, their expression levels did not always show a high AUC for the prediction of tumor prognosis (Figure S1). The importance of out-of-bag of these 5 genes was ranked and is shown in Figure 3(b). A 5-gene signature was established using multivariate Cox regression analysis, and the model is as follows:

(2) $$\begin{array}{c} {\text{Risk}}_{5}=-0.3337301*\text{HIST}12\text{BH}+0.2931728*\text{SERPIND}1+0.2956749*\text{COL}22\text{A}1+0.2592219*\text{LCE}3\text{C}-0.2458371*\text{ADAMTS}17. \end{array}$$

The risk score of each sample was calculated, and the samples were grouped according to the mid-value of the risk score (cutoff = ‐0.05950035). A significant difference in prognosis, which is a carcinogenic signature, was identified between the high-risk group and the low-risk group (Figure 3(c)). The 3-year AUC of the 5-gene signature in the training set was 0.76 (Figure 3(d)). The relationship between the expressions of the 5 genes and risk score was observed; specifically, high expressions of SERPIND1, COL22A1, and LCE3C were found correlated with a high risk, and these genes are therefore considered risk factors, while highly expressed HIST1H2BH and ADAMTS17 were correlated with a low risk and could be regarded as protective factors.

### 3.5. Verification of the Robustness of the 5-Gene Signature Model

**Figure 4:**
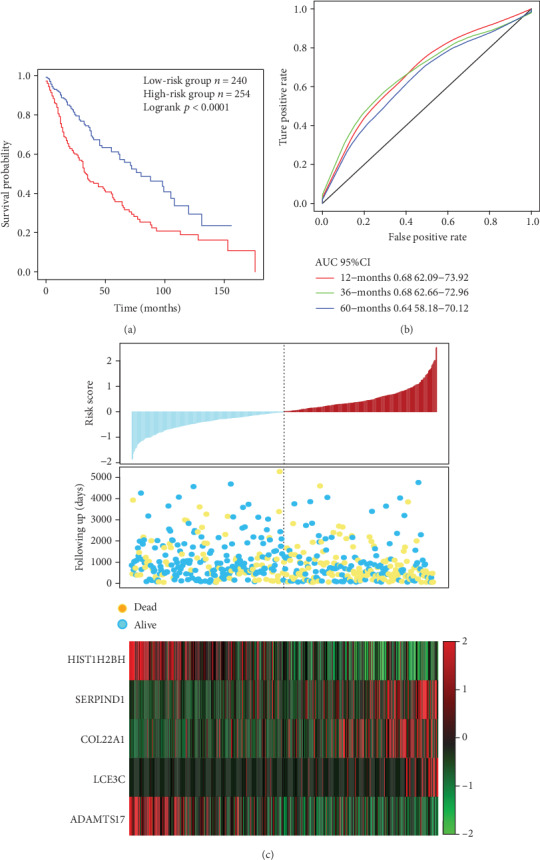
(a) Distribution of 5-gene signature's Kaplan-Meier (KM) survival curve in the TCGA test. (b) ROC curve and AUC of the 5-gene signature classification. (c) TCGA test focused on risk score, survival time and survival status, and the expressions of 5 genes.

In order to determine the robustness of the 5-gene signature model, the risk score of each sample was calculated in the test set, and the samples were divided into two groups according to the threshold of the training set, with significant prognostic differences observed between the two groups (Figure 4(a)). ROC analysis showed that the 5-year AUC reached 0.68 (Figure 4(b)). Furthermore, the analysis of the relationship between the expressions of the 5 genes and risk score revealed that SERPIND1, COL22A1, and LCE3C were associated with a high risk and were seen as risk factors, while HIST1H2BH and ADAMTS17 were indicative of low risk; thus, the two could serve as protective factors. This is also consistent with the training set results (Figure 4(c)). In conclusion, the model showed highly effective prognosis classification results in the TCGA dataset.

**Figure 5:**
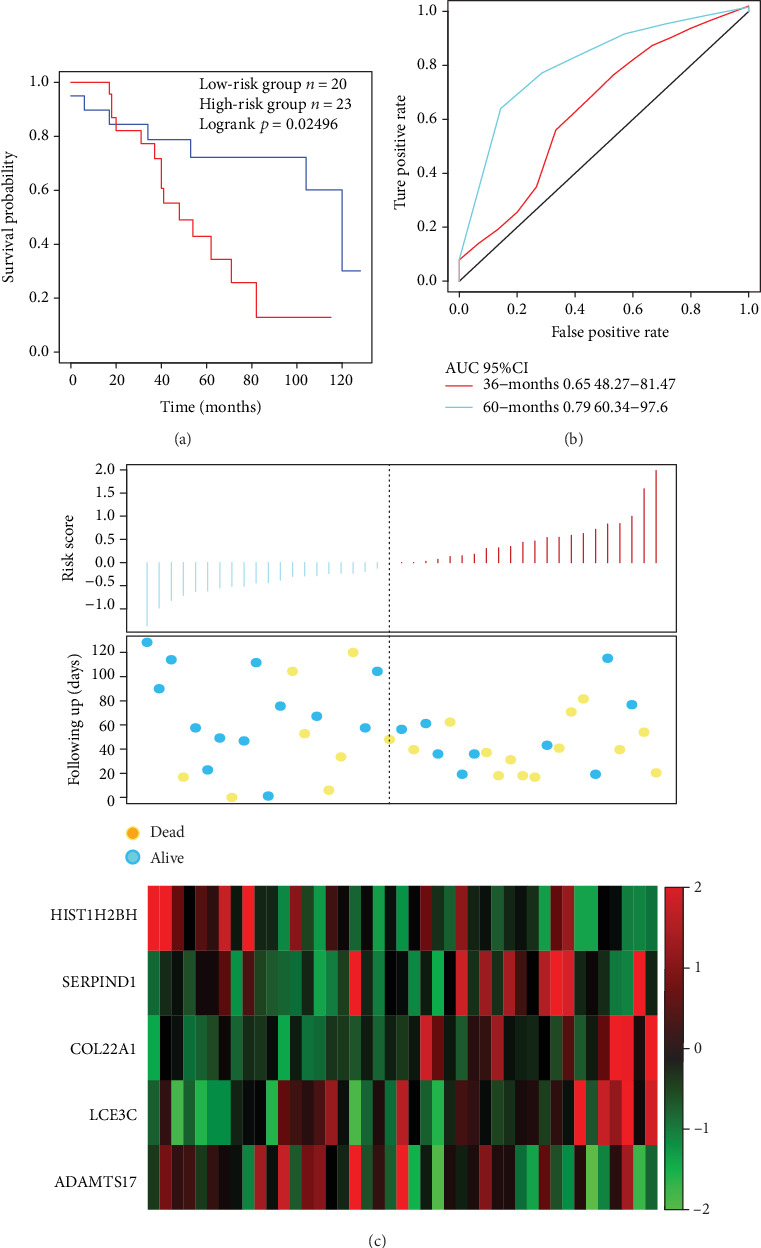
(a) The 5-gene signature's KM survival curve distribution in GSE42127. (b) ROC curve and AUC of the 5-gene signature classification. (c) Risk score, survival time, survival status, and expression of 5 genes in GSE42127.

**Figure 6:**
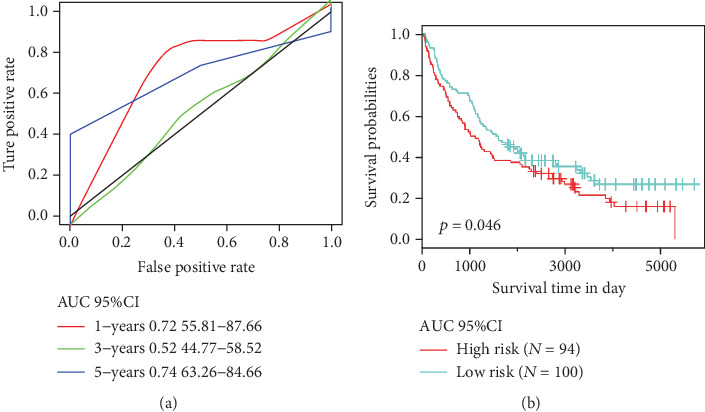
(a) ROC curve and AUC of the 5-gene signature in GSE37745. (b) risk score, survival time, survival status, and expressions of the 5 genes in GSE37745.

In order to verify the classification performance of the 5-gene signature model in data from different data platforms, GEO platform data GSE42127 and GSE37745 were taken as the external dataset. The signature model was used to calculate the risk score of each sample, and the cutoff of the training set was used to divide the samples into the high-risk and low risk groups. The results demonstrated that the prognosis of the low-risk group was significantly better than that of the high-risk group (Figure 5(a)). Moreover, ROC analysis showed that the 3-year AUC reached 0.79 (Figure 5(b)). The relationship between the expressions of 5 genes and the risk score was also consistent with the training set (Figure 5(c)). In addition, the similar results were observed in the GSE37745 dataset, 5-year AUC was found to be 0.74, and the OS between the two groups showed a significant difference (Figure 6). In conclusion, our 5-gene signature model has demonstrated its predictive performance of prognosis for both internal and external datasets.

### 3.6. Clinical Independence of the 5-Gene Signature Model

In order to identify the independence of the 5-gene signature model in clinical application, univariate and multivariate Cox regression analyses were performed to analyze the relevant HR, 95% CI of HR, *p* value of the TCGA training set, TCGA dataset, and GSE42127 data. Clinical information and our 5-gene signature grouped information, including age; sex; smoking history; pathology stages T, N, and M; and tumor stage, were systematically analyzed from TCGA and GSE42127 patients (Table 4). In the TCGA training set, univariate Cox regression analysis found that the high-risk group, pathologic T4, and pathologic M1/MX were significantly correlated with OS; however, the corresponding multivariate Cox regression analysis revealed that only the high risk group (HR = 2.27, 95%CI = 1.30601-3.936, *p* = 0.004) had clinical independence. In the TCGA dataset, the univariate Cox regression analysis found that the high-risk group, pathologic T3, pathologic T4, pathologic M1, and tumor stage III were greatly associated with OS, whereas the corresponding multivariate Cox regression analysis demonstrated that only the high-risk group (HR = 1.737, 95% CI = 1.2456-2.423, *p* = 1.14*E* − 03) and pathologic MX (HR = 2.250, 95% CI = 1.1797-4.293, *p* = 0.014) were clinically independent. In conclusion, our model 5-gene signature is a prognostic indicator independent of other clinical factors and has independent predictive performance with a clinical application value.

### 3.7. Comparison of the 5-Gene Signature Model with Other Models

**Figure 7:**
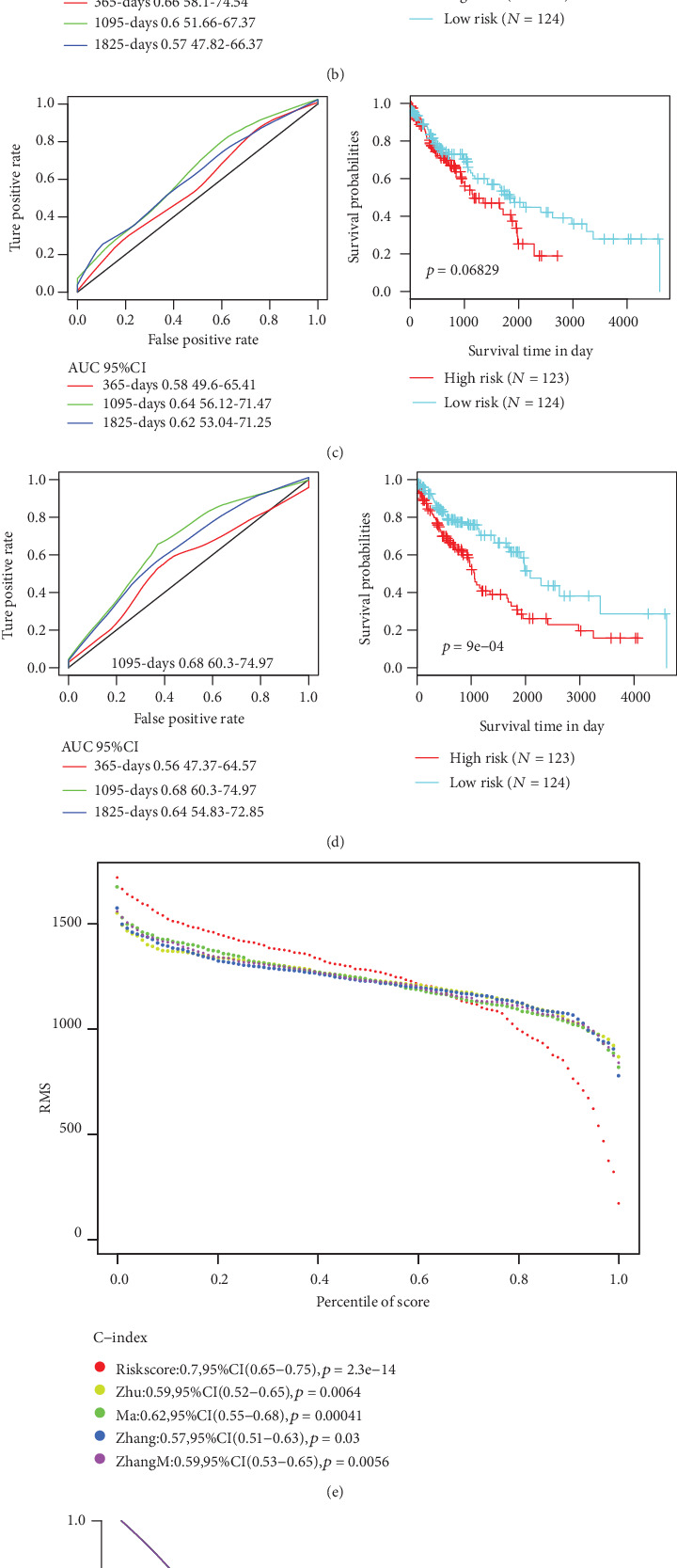
Comparison and analysis of the 5-gene signature model and other existing models. (a) AUC and KM curves of autophagy-related gene prognostic signature by Zhu et al. (b) AUC and KM curves of immune-related signature by Zhang et al. (c) AUC and KM curves of sixteen-gene prognostic biomarker by Zhang et al. (d) AUC and KM curves of glycolysis-related gene signature by Zhang et al. (e) RMS curves of four models and the 5-gene signature. (f) DCA curves of four models and the 5-gene signature.

The performance of the 5-gene signature model was compared with other 4 previously established prognostic feature signatures, namely, autophagy-related gene prognostic signature by Zhu et al., sixteen-gene prognostic biomarker by Ma et al., glycolysis-related gene signature by Zhang et al., and immune-related signature by Zhang et al. In order to allow those models to be more comparable, we calculated the risk score of each sample in the TCGA using the same method based on the corresponding genes in the 4 models. The ROC of each model was examined, and the samples were divided into the high-risk and low-risk groups based on the median risk score, and the OS prognosis difference between the two groups of samples was calculated. The KM curve of OS showed that the prediction performance of the four models was less accurate than our 5-gene signature model (Figures 7(a)–7(d)). To further compare the predictive performance of these models on TCGA samples, the “rms” package in R was used to calculate the restricted mean survival curves of the 4 models and our model, and the results demonstrated that our model has the highest C-index among the total 5 models investigated (Figure 7(e)); noticeably, our 5-gene model also showed more advantages in long-term survival prediction. Furthermore, we compared the 5-gene signature and the prediction results of the 4 models by the DCA curve, and results showed that the performance of the 5-genes signature model established in the current study was higher than those of the other four (Figure 7(f)).

### 3.8. GSEA Analysis on Enriched Pathway Differences between the High-Risk Group and the Low-Risk Group

**Figure 8:**
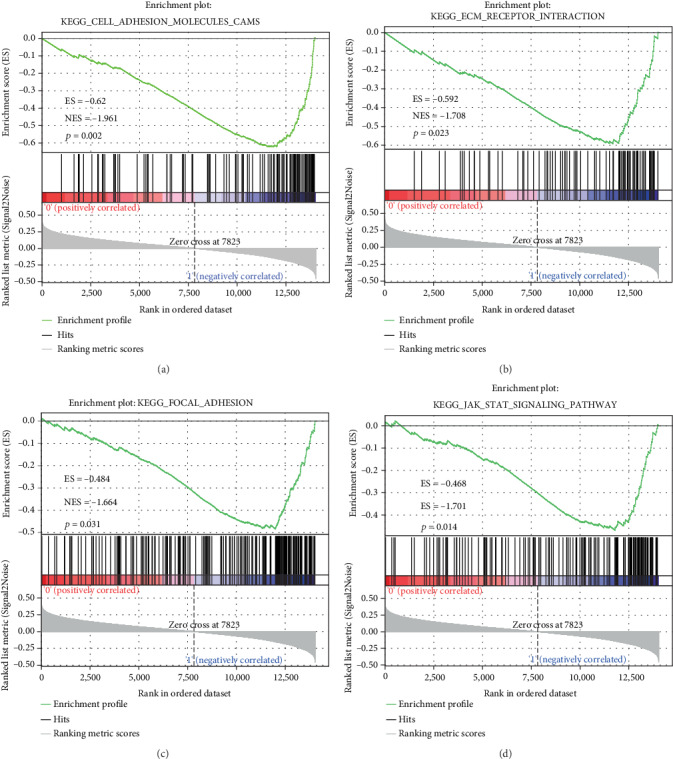
Five-gene signature-enriched pathways in high-risk and low-risk groups.371

In the TCGA training set, GSEA used to identify the significantly enriched pathways in the high-risk group and the low-risk group screened a total of 41 significantly enriched pathways (Table 5). Among them, KEGG cell adhesion molecules (cams), KEGG ECM receptor interaction, KEGG JAK STAT signaling pathway, and KEGG focal adhesions were all significantly related to the occurrence, development, and metastasis of LSCC (Figure 8).

## 4. Discussion

Lung cancer is a leading cause of cancer deaths, and the incidence of the cancer is increasing worldwide. In all lung cancer cases, LSCC causes an annual death of at least 400,000. Similar to many other cancers, lung cancer patients are usually at advanced stages by the time of diagnosis, suggesting that there is nearly no available treatment for the patients. However, early diagnosis and surgical resection can significantly improve the survival rate of lung cancer patients. The development of molecular biomarkers play an important role in personalized medicine and current precision medicine [27]; therefore, there is an urgent need for a classifier for predicting the prognosis of LSCC patients with poor prognosis and designing customized therapies. In our current study, transcriptome, copy number variation, and mutation data were mined from TCGA to search for obtaining novel prognostic markers for LSCC. Interestingly, we found that the gene signature constructed from 5 differentially expressed genes demonstrated great prediction performance for LSCC.

TCGA data with large-scale genome analysis allowed it to be possible to examine the molecular characteristics associated with LSCC results [28]. In 2015, Huang et al. analyzed gene and miRNA expressions, DNA methylation, and CNV data of 129 LSCC specimens in TCGA, and they established a genome-wide integration network by using variance expansion factor regression and isolated lung cancer subnetwork by the Bayesian method [29]. LSCC patients with a 4-gene expression signature among 14 differentially expressed feature genes were at a high risk of developing a poor prognosis. Gao et al. also reported that 12 of the 133 abnormally expressed miRNAs were correlated with OS in the TCGA LSCC cohort [30]. The AUC of our 5-gene signature was close to 0.7 in the training set, test set, and verification set. All these genes had abnormal genome mutations, which allows an easy clinical detection. In a word, our 5-gene signature had high AUC and involved fewer genes; thus, it was conducive to clinical transformation.

We were also interested in investigating the prospective molecular mechanisms of these 5 genes. Therefore, GSEA analysis was conducted to explore related gene enrichment pathways. SERPIND1, COL22A1, and LCE3C are risk factors, while ADAMTS17 is a protective factor in gene signature. Hereinto, SERPIND1 acts as a potential oncogene in the development of tumor, including in lung cancer [31, 32]. In head and neck cancer, highly expressed COL22A1 mRNA is statistically correlated with reduced disease-free survival and is significantly associated with lymph node metastasis [33]. HIST1H2BH is associated with the prognosis of cervical cancer patients [34]. Knocking down Adamts17 expression induces the apoptosis of breast cancer cells and inhibits cancer cell growth [35]. However, LCE3C has not been shown to be associated with tumor. In this study, for the first time, LCE3C was found to be a new prognostic marker for lung adenocarcinoma. Meanwhile, our GSEA analysis results showed that the pathway enriched by the 5-gene signature was significantly correlated with the pathway and biological process of the occurrence and development of LSCC. These results indicated that our 5-gene model has a potential clinical application value and could provide a potential target for the clinical diagnosis of LSCC patients.

It should be noted that though potential candidate genes for tumor prognosis were identified in large samples through bioinformatics techniques, some limitations still exist in the present research. Firstly, the sample lacked certain clinical follow-up information; thus, we did not consider factors such as the presence of other health statuses of the patient in distinguishing prognostic biomarkers. Second, the results obtained only through bioinformatics analysis are not fully adequate; thus, experimental validation is required to further confirm our findings. Moreover, validation and experimental studies should be conducted on a larger sample size.

## 5. Conclusion

In conclusion, in this study, a 5-gene signature prognostic stratification system has been developed, and the model demonstrated great AUC in both the training set and validation set and was independent of clinical features. Compared with clinical features, gene classifier can improve the prediction of survival risk. Therefore, this classifier could serve as a molecular diagnostic test in the evaluation of the prognostic risk of patients with LSCC.

## Figures and Tables

**Table 1:** Clinical information statistics of three sets of datasets.

Characteristic		TCGA training datasets (*n* = 247)	TCGA all datasets (*n* = 494)	GSE42127 (*n* = 43)
Age (years)	≤50	8	19	0
	>50	238	470	43

Survival status	Living	144	282	22
	Dead	103	212	21

Gender	Female	62	128	18
	Male	185	366	25

Smoke years	≤20	11	21	
	>20	94	199	

pathologic_T	T 1	54	114	
	T 2	146	287	
	T 3	36	70	
	T 4	11	23	

pathologic_N	N 0	158	316	
	N 1	67	127	
	N 2	18	40	
	N 3	1	5	

pathologic_M	M 0	194	406	
	M 1/M X	50	84	

Tumor stage	Stage I	120	242	23
	Stage II	81	158	10
	Stage III	41	83	10
	Stage IV	4	7	

**Table 2:** Top 20 prognosis-related gene information.

ENSG ID	HR	Coefficient	*Z*-score	*p* value
ENSG00000229859	1.381	0.323	4.186	2.84*E*-05
ENSG00000133055	1.293	0.257	4.070	4.71*E*-05
ENSG00000249158	1.386	0.327	3.952	7.74*E*-05
ENSG00000100632	0.645	-0.439	-3.904	9.48*E*-05
ENSG00000188467	1.456	0.376	3.821	0.000132923
ENSG00000080511	1.283	0.249	3.794	0.000148025
ENSG00000069509	0.673	-0.396	-3.780	0.000156912
ENSG00000187733	1.265	0.235	3.759	0.000170307
ENSG00000072657	1.348	0.298	3.730	0.000191851
ENSG00000099937	1.341	0.293	3.718	0.000200903
ENSG00000126752	1.267	0.237	3.706	0.000210942
ENSG00000179520	1.244	0.218	3.702	0.000213691
ENSG00000100994	1.430	0.358	3.462	0.000535801
ENSG00000041353	1.409	0.343	3.448	0.0005653
ENSG00000162551	1.412	0.345	3.392	0.000694076
ENSG00000271447	1.328	0.284	3.383	0.000717711
ENSG00000165762	1.239	0.214	3.356	0.000791736
ENSG00000172789	1.306	0.267	3.335	0.000852719
ENSG00000105650	1.276	0.244	3.306	0.000947676
ENSG00000253537	1.371	0.316	3.305	0.000949615

**Table 3:** Five genes significantly associated with the overall survival in the training-set patients.

Ensembl gene ID	Symbol	HR	*Z*-score	*p* value	Importance	Relative importance
ENSG00000275713	HIST1H2BH	0.69	-3.239003	1.20*E*-03	0.0178	1
ENSG00000099937	SERPIND1	1.34	3.717879	2.01*E*-04	0.0115	0.648
ENSG00000169436	COL22A1	1.29	2.732945	6.28*E*-03	0.0105	0.593
ENSG00000244057	LCE3C	1.26	3.234898	1.22*E*-03	0.0093	0.5216
ENSG00000140470	ADAMTS17	0.74	-2.686823	7.21*E*-03	0.0049	0.2729

**Table 4:** Univariate and multivariate Cox regression analysis to identify prognostic clinical factors and clinical independence.

Variables	Univariate analysis			Multivariable analysis		
	HR	95% CI of HR	*p* value	HR	95% CI of HR	*p* value
TCGA training datasets						
5-gene risk score						
Low-risk group	1 (reference)			1 (reference)		
High-risk group	2.72	2.01-3.66	5.310*E*-11	2.27	1.30601-3.936	0.004
Age	1.00	0.97-1.02	9.980*E*-01	1.03	0.97001-1.093	0.33624
Gender female	1 (reference)			1 (reference)		
Gender male	0.92	0.58-1.43	0.70	0.63	0.30321-1.301	0.21085
Smoke years	0.98	0.96-1.01	0.37	0.99	0.96278-1.015	0.40262
Pathologic T1	1 (reference)			1 (reference)		
Pathologic T2	1.41	0.8314-2.381	0.20341	1.48	0.52948-4.114	0.45674
Pathologic T3	1.69	0.8693-3.283	0.122	1.74	0.2188-13.78	0.602
Pathologic T4	3.90	1.4234-10.69	0.008	13.87	0.25483-754.666	0.197
Pathologic N0	1 (reference)			1 (reference)		
Pathologic N1	1.21	0.7879-1.857	0.384	0.54	0.10297-2.854	0.470
Pathologic N2/N3	0.77	0.3517-1.686	0.513	0.91	0.02173-37.89	0.959
Pathologic M0	1 (reference)			1 (reference)		
Pathologic M1/MX	1.70	1.059-2.717	2.80E-02	3.65	1.58507-8.395	2.34*E*-03
Tumor stage I	1 (reference)			1 (reference)		
Tumor stage II	1.02	0.6474-1.615	0.924	1.46	0.32211-6.599	0.62452
Tumor stage III	1.27	0.7519-2.132	0.3749	0.89	0.0297-26.834	0.94791
Tumor stage IV	3.23	0.9983-10.429	0.050	1.31	0.08057-21.187	0.85

*TCGA datasets, GSE42127*						
*TCGA test datasets*						
5-gene risk score						
Low-risk group	1 (reference)			1 (reference)		
High-risk group	1.85	1.501-2.283	8.89*E*-09	1.737	1.2456-2.423	1.14*E*-03
Age	1.02	0.9995-1.033	0.058	1.029	0.9916-1.067	0.132
Gender female	1 (reference)			1 (reference)		
Gender male	1.20	0.8669-1.646	0.277	1.240	0.7443-2.067	0.408
Smoke years	0.99	0.9739-1.007	0.26	0.99	0.973-1.01	0.349
Pathologic T1	1 (reference)			1 (reference)		
Pathologic T2	1.25	0.8779-1.765	0.219	0.89	0.4878-1.629	0.708
Pathologic T3	1.82	1.1618-2.847	0.009	0.897	0.27-2.98	0.859
Pathologic T4	2.32	1.2481-4.327	0.008	1.014	0.2338-4.396	0.985
Pathologic N0	1 (reference)			1 (reference)		
Pathologic N1	1.07	0.7824-1.466	0.669	0.687	0.2744-1.721	0.423
Pathologic N2	1.32	0.831-2.093	2.40*E*-01	1.475	0.3304-6.588	0.611
Pathologic N3	2.51	0.6183-10.212	1.98*E*-01	3.030	0.3645-25.18	0.305
Pathologic M0	1 (reference)			1 (reference)		
Pathologic M1	3.18	1.3-7.778	1.13*E*-02	1.472	0.1424-15.22	0.746
Pathologic MX	1.55	1.049-2.299	0.028	2.250	1.1797-4.293	0.014
Tumor stage	1 (reference)			1 (reference)		
Tumor stage II	1.13	0.8234-1.559	4.43*E*-01	1.011	0.4121-2.48	0.981
Tumor stage III	1.64	1.1622-2.311	4.84*E*-03	1.351	0.2684-6.796	0.716

*GSE42127*						
5-gene risk score						
Low risk group	1 (reference)			1 (reference)		
High risk group	2.07	1.133-3.778	0.018	2.33	1.1539-4.697	0.018
Age	1.03	0.9751-1.084	0.306	1.0127	0.9497-1.08	0.7002
Gender female	1 (reference)			1 (reference)		
Gender male	1.196	0.4723-3.029	0.706	1.1037	0.4032-3.021	0.848
Tumor stage I	1 (reference)			1 (reference)		
Tumor stage II	0.82	0.2549-2.643	0.741	1.00	0.2773-3.576	0.995
Tumor stage III	2.0311	0.7574-5.447	0.159	2.7395	0.9225-8.136	0.070

**Table 5:** GSEA analyzed significantly enriched KEGG pathways in high-risk and low-risk groups.

Name	Size	ES	NES	NOM *p*-val	FDR *q*-val	FWER *p*-val
KEGG_CYTOKINE_CYTOKINE_RECEPTOR_INTERACTION	243	-0.609	-2.002	0.0001	0.055	0.037
KEGG_LEISHMANIA_INFECTION	64	-0.709	-1.985	0.0001	0.039	0.052
KEGG_COMPLEMENT_AND_COAGULATION_CASCADES	68	-0.726	-1.969	0.0001	0.035	0.064
KEGG_HEMATOPOIETIC_CELL_LINEAGE	84	-0.708	-1.967	0.0001	0.026	0.064
KEGG_CELL_ADHESION_MOLECULES_CAMS	122	-0.623	-1.961	0.002	0.023	0.07
KEGG_VIRAL_MYOCARDITIS	67	-0.636	-1.933	0.0001	0.028	0.1
KEGG_LEUKOCYTE_TRANSENDOTHELIAL_MIGRATION	108	-0.551	-1.920	0.002	0.029	0.115
KEGG_AUTOIMMUNE_THYROID_DISEASE	49	-0.710	-1.817	0.002	0.075	0.259
KEGG_CHEMOKINE_SIGNALING_PATHWAY	178	-0.491	-1.799	0.020	0.080	0.3
KEGG_ASTHMA	27	-0.790	-1.795	0.0001	0.075	0.306
KEGG_TYPE_I_DIABETES_MELLITUS	40	-0.747	-1.791	0.008	0.070	0.316
KEGG_GLYCOSPHINGOLIPID_BIOSYNTHESIS_GANGLIO_SERIES	14	-0.681	-1.732	0.008	0.107	0.454
KEGG_INTESTINAL_IMMUNE_NETWORK_FOR_IGA_PRODUCTION	45	-0.727	-1.720	0.009	0.109	0.476
KEGG_NATURAL_KILLER_CELL_MEDIATED_CYTOTOXICITY	127	-0.510	-1.710	0.018	0.112	0.505
KEGG_ALLOGRAFT_REJECTION	34	-0.807	-1.709	0.006	0.105	0.508
KEGG_ECM_RECEPTOR_INTERACTION	82	-0.592	-1.708	0.023	0.100	0.511
KEGG_JAK_STAT_SIGNALING_PATHWAY	147	-0.468	-1.701	0.014	0.100	0.525
KEGG_ANTIGEN_PROCESSING_AND_PRESENTATION	78	-0.581	-1.692	0.033	0.102	0.546
KEGG_LYSOSOME	115	-0.483	-1.683	0.024	0.103	0.562
KEGG_GRAFT_VERSUS_HOST_DISEASE	36	-0.780	-1.669	0.016	0.109	0.595
KEGG_FOCAL_ADHESION	189	-0.484	-1.664	0.031	0.109	0.607
KEGG_PRION_DISEASES	35	-0.487	-1.662	0.006	0.106	0.613
KEGG_RENIN_ANGIOTENSIN_SYSTEM	16	-0.603	-1.655	0.028	0.107	0.624
KEGG_NOD_LIKE_RECEPTOR_SIGNALING_PATHWAY	54	-0.489	-1.608	0.041	0.142	0.722
KEGG_HISTIDINE_METABOLISM	26	-0.484	-1.592	0.046	0.154	0.757
KEGG_HYPERTROPHIC_CARDIOMYOPATHY_HCM	82	-0.445	-1.583	0.037	0.157	0.776
KEGG_TOLL_LIKE_RECEPTOR_SIGNALING_PATHWAY	99	-0.441	-1.574	0.048	0.160	0.787
KEGG_OTHER_GLYCAN_DEGRADATION	15	-0.587	-1.532	0.060	0.201	0.852
KEGG_PATHOGENIC_ESCHERICHIA_COLI_INFECTION	47	-0.415	-1.515	0.026	0.216	0.874
KEGG_BASAL_TRANSCRIPTION_FACTORS	34	0.584	1.895	0.004	0.141	0.131
KEGG_NUCLEOTIDE_EXCISION_REPAIR	44	0.607	1.855	0.004	0.124	0.2
KEGG_CELL_CYCLE	112	0.531	1.841	0.004	0.100	0.231
KEGG_HOMOLOGOUS_RECOMBINATION	24	0.700	1.831	0.004	0.082	0.246
KEGG_SPLICEOSOME	90	0.605	1.811	0.004	0.081	0.283
KEGG_DNA_REPLICATION	32	0.732	1.767	0.004	0.106	0.392
KEGG_MISMATCH_REPAIR	23	0.674	1.718	0.016	0.141	0.503
KEGG_BASE_EXCISION_REPAIR	33	0.609	1.680	0.024	0.165	0.59
KEGG_RNA_DEGRADATION	47	0.530	1.676	0.012	0.152	0.6
KEGG_GLYCOSYLPHOSPHATIDYLINOSITOL_GPI_ANCHOR_BIOSYNTHESIS	22	0.570	1.638	0.024	0.179	0.675
KEGG_RNA_POLYMERASE	28	0.556	1.622	0.033	0.181	0.711

## Data Availability

The data used to support the findings of this study are available from the corresponding author upon request.

## Supplementary Materials

Supplementary Materials These genes play important roles in the regulation of tumor-related pathways and biological processes; however, their expression levels did not always show a high AUC for the prediction of tumor prognosis (Figure S1).
