# Supplementary figures and images for: Identification of 5 Gene Signatures in Survival Prediction for Patients with Lung Squamous Cell Carcinoma Based on Integrated Multiomics Data Analysis
Source: Biomed Res Int. 2020 Jun 8;2020:6427483. doi: 10.1155/2020/6427483 (PMC7298313; doi:10.1155/2020/6427483)

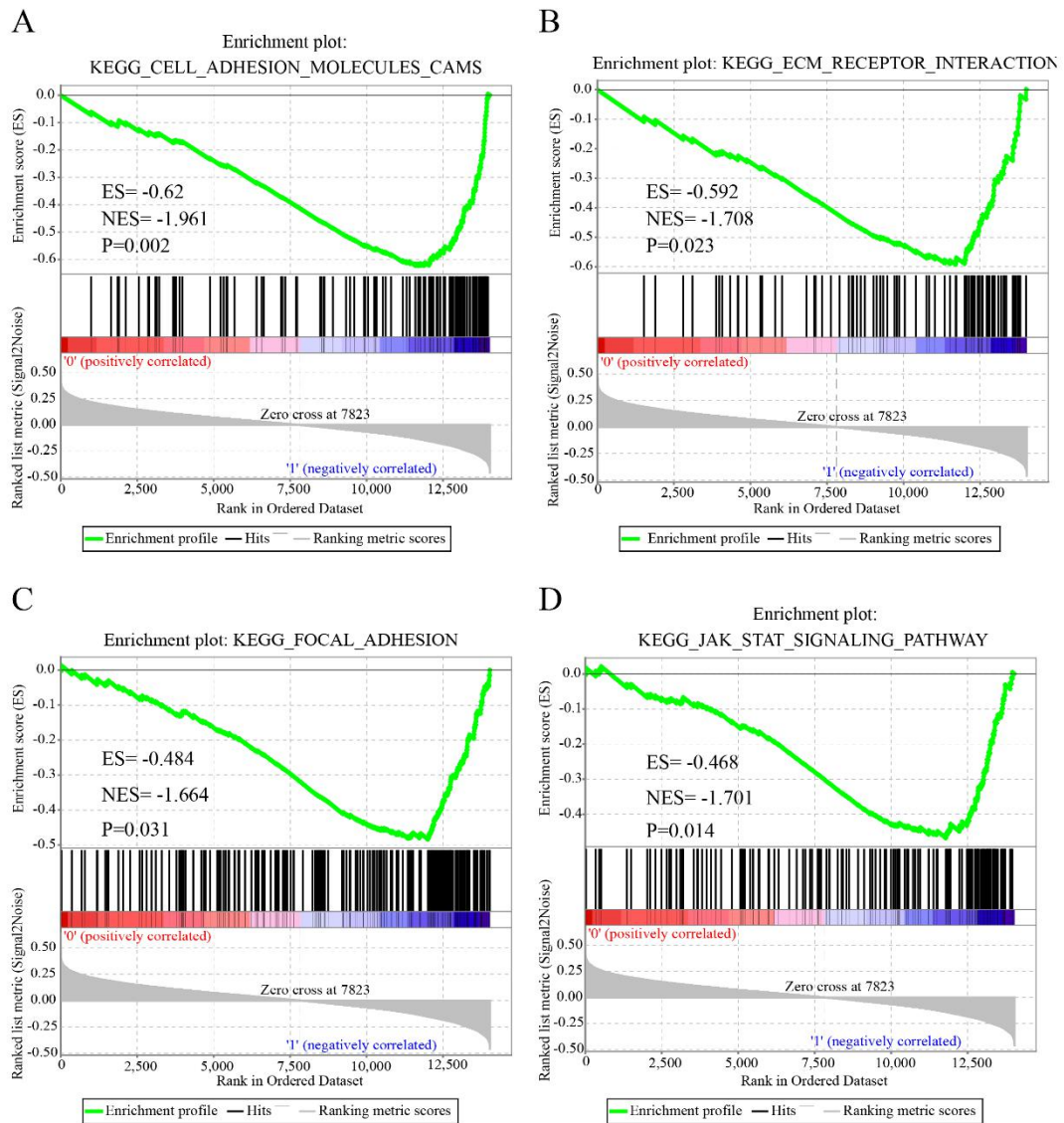

**Figure S1**

Supplement: Supplementary Materials — These genes play important roles in the regulation of tumor-related pathways and biological processes; however, their expression levels did not always show a high AUC for the prediction of tumor prognosis (Figure S1). [file 6427483.pdf]
